# Supplementary material for: Low-N Protein Activity Optimization with FolDE
Source: ArXiv. 2025 Oct 28:arXiv:2510.24053v1. Preprint. [Version 1] (PMC12636759)
Supplement: Supplement 1 [file NIHPP2510.24053v1-supplement-1.pdf]

Supplementary Information

Table S1: ProteinGym Datasets Used in Study

Set	ProteinGym ID	Number of Mutants	Notes
Train	ANCSZ_Hobbs_2022	4670	
Train	BLAT_ECOLX_Firnberg_2014	4783	
Train	CBS_HUMAN_Sun_2020	7217	
Train	HEM3_HUMAN_Loggerenberg_2023	5689	
Train	HSP82_YEAST_Flynn_2019	13294	
Train	hKKA_HUMAN_Gersing_2022_activity	8570	
Train	OXDA_RHOTO_Vanella_2023_activity	6396	
Train	PPM1D_HUMAN_Miller_2022	7889	
Train	SHOQ2_HUMAN_Kwon_2022	10972	
Single Mutation	ADGRB6_PSEA1_Chen_2020	5004	
Single Mutation	AMIE_PSEAE_Wrenbeck_2017	6227	
Single Mutation	CAS9_STRP1_Spencer_2017_positive	8117	
Single Mutation	HMDH_HUMAN_Jiang_2019	16853	
Single Mutation	KCNJ2_MOUSE_Coyote-Maestas_2022_function	6963	
Single Mutation	KKAA_KLEPN_Melnikov_2014	4960	
Single Mutation	LGK_LIPST_Klesmith_2015	7890	
Single Mutation	MET_HUMAN_Eastew_2023	5393	
Single Mutation	MLAC_ECOLI_MacRae_2023	4007	
Single Mutation	MSH2_HUMAN_Jia_2020	16749	
Single Mutation	MTHR_HUMAN_Weile_2021	12464	
Single Mutation	PAH_HUMAN_Huttinger_2021	5345	
Single Mutation	PPARG_HUMAN_Majithia_2016	9576	
Single Mutation	PTEN_HUMAN_Mighell_2018	7260	
Single Mutation	RNC_ECOLI_Weeks_2023	4277	
Single Mutation	S22A1_HUMAN_Yee_2023_activity	1094	
Single Mutation	SC6A4_HUMAN_Young_2021	11576	
Multiple Mutation	GRB2_HUMAN_Faure_2021	63366	120 double mutants per single mutant
Multiple Mutation	PABP_YEAST_Melamed_2013	37708	61 double mutants per single mutant
Multiple Mutation	SPG1_STRSG_Olson_2014	536962	1026 double mutants per single mutant

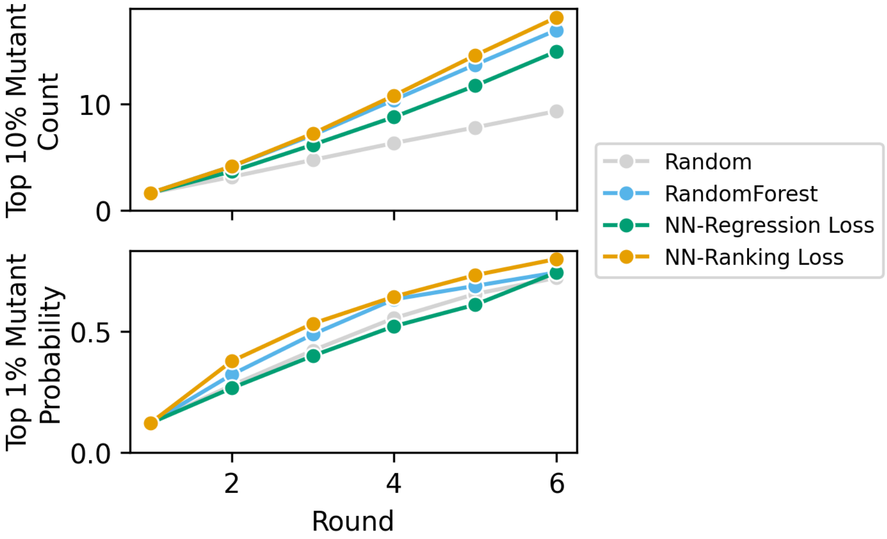

Figure S1: **Top Layer Architectures** Prediction quality of three top-layer architectures: random forest, a neural network trained with mean squared error loss, and a neural network trained with ranking loss. Evaluated on the training benchmark. All have random mutants selected in round-1.

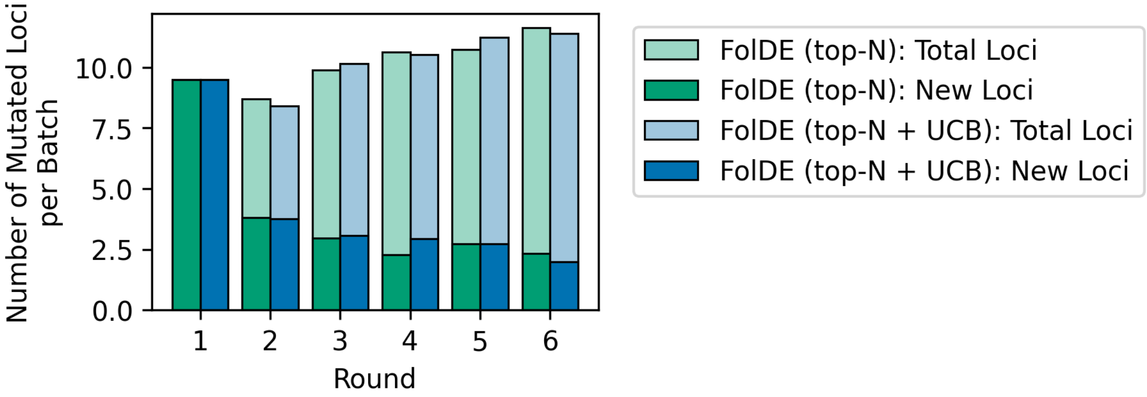

Figure S2: **Upper Confidence Bound (UCB) Does Not Improve Batch Diversity** The number of mutated loci per batch on the multi-mutation benchmark decreases over rounds for both standard top-N selection and top-N with UCB. Dark bars show new loci not previously mutated; light bars show previously mutated loci. Both selection methods show identical patterns of declining exploration, with the majority of later-round mutations concentrated on previously successful loci. Data averaged across multi-mutation benchmark proteins.

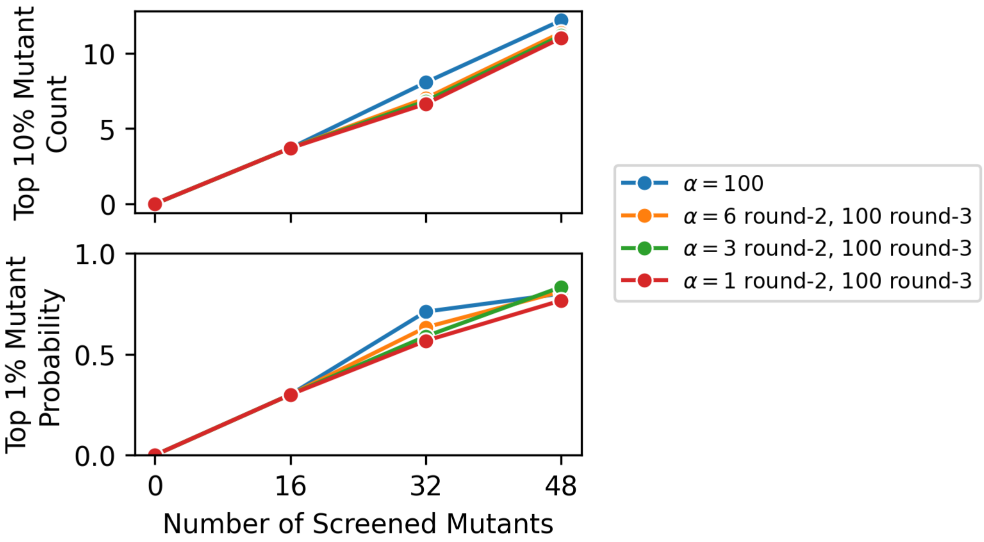

Figure S3: **Optimizing Constant-Liar α For Three Round Campaign** More aggressive constant-liar (lower α) worsens the number of top 10% mutants discovered and, for some values, improves the 3-round probability of finding a top 1% mutant.

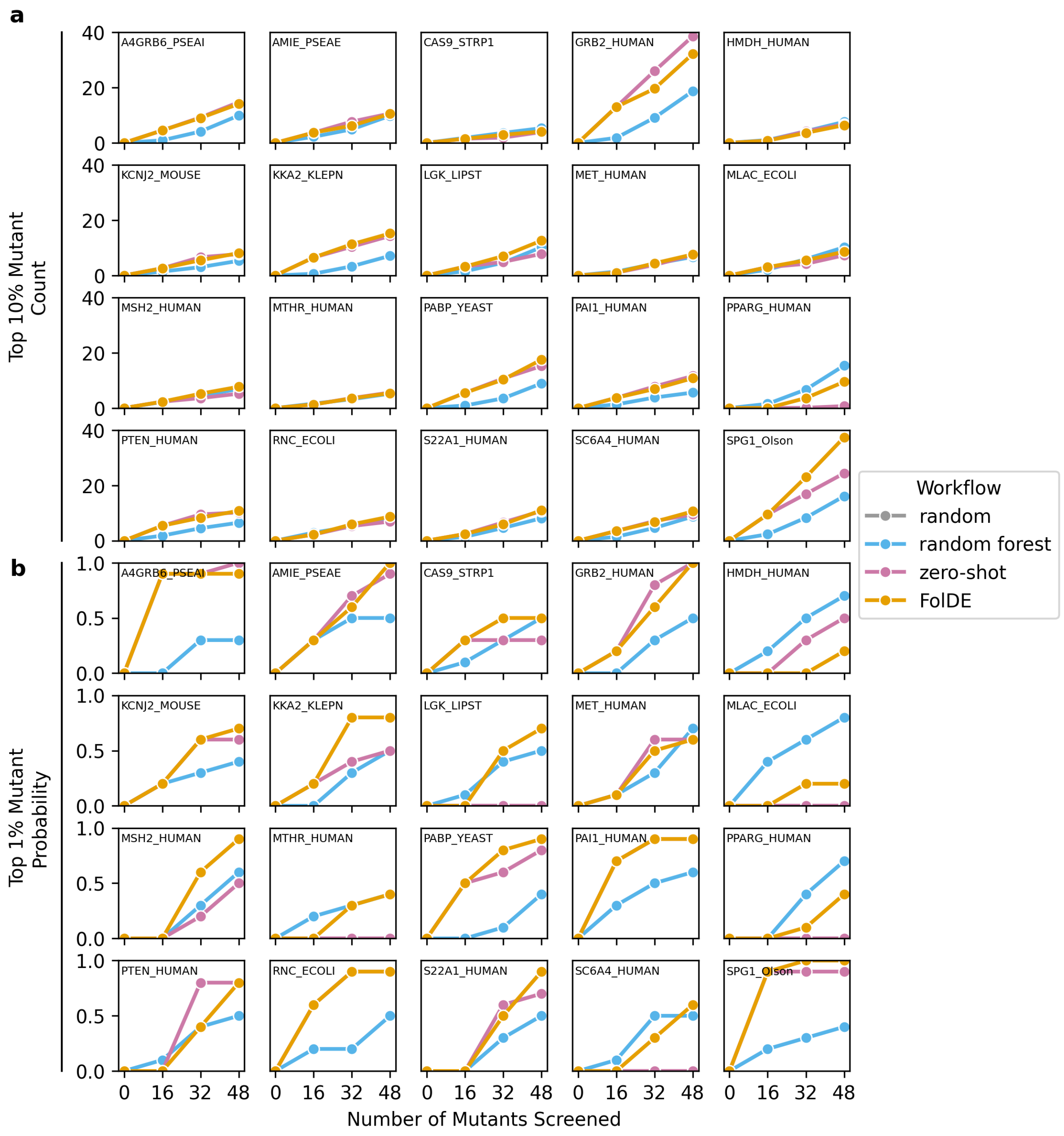

Figure S4: **FolDE Comparison to Baseline, Per-Target, for a Three Round Campaign** (a) the number of top 10% performing mutants discovered and (b) probability of finding a top 1% mutant.

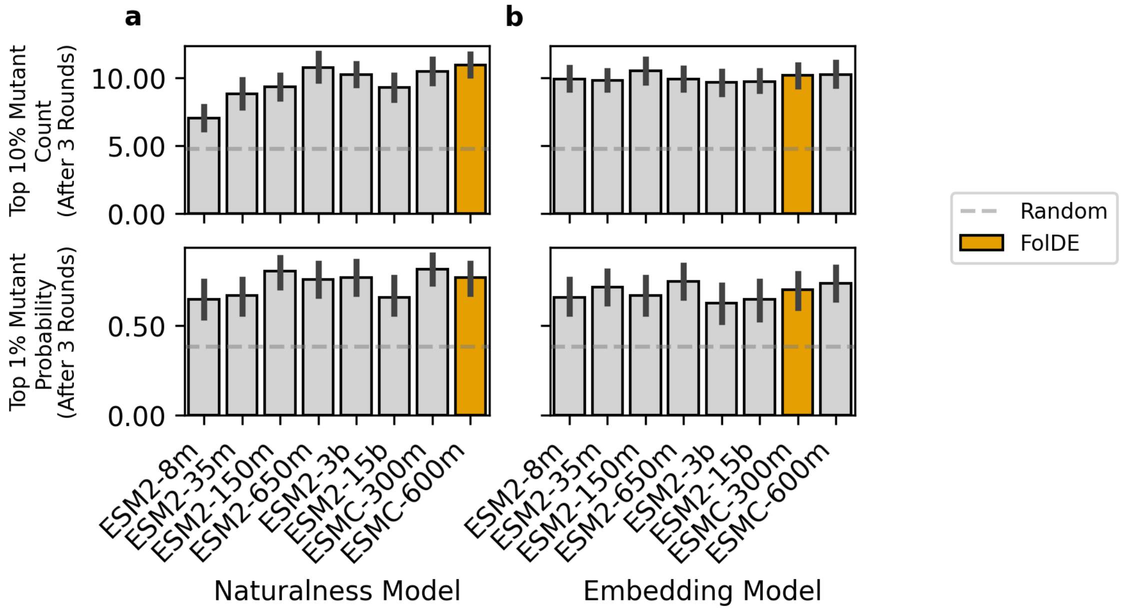

Figure S5: **Protein Language Model Sizing** (a) Prediction quality of the PLM as a zero-shot activity predictor was evaluated on the training set of proteins, as measured by both the cumulative number of top 10% mutants discovered after 3 rounds (top) and the probability of finding a top 1% mutant (bottom). (b) Similarly, the performance of the FolDE model was evaluated on round-3 for different PLM embedding models.

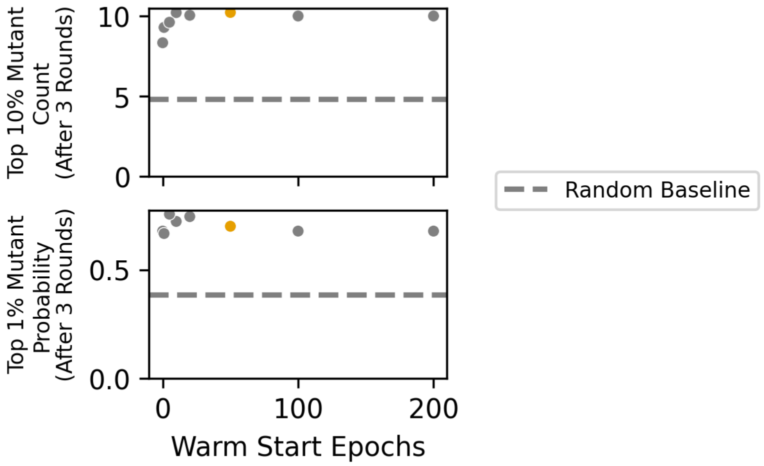

Figure S6: **Calibrating the Naturalness Warm-Start** For round-3 evaluated on the training dataset, a sweep over warm-start training epochs shows that the improvements have stabilized after about 10 epochs, as measured by both cumulative top 10% mutants and probability of finding a top 1% mutant. Evaluated on 0, 1, 5, 10, 20, 50, 100, 200 epochs.
